# Acceptability of Delivering and Accessing Health Information Through Text Messaging Among Community Health Advisors

**DOI:** 10.2196/mhealth.2641

**Published:** 2013-09-09

**Authors:** Yu-Mei Schoenberger, Janice Phillips, Mohammed Omar Mohiuddin, Patrick McNees, Isabel Scarinci

**Affiliations:** ^1^Division of Preventive MedicineUniversity of Alabama at BirminghamBirmingham, ALUnited States; ^2^School of Health ProfessionsUniversity of Alabama at BirminghamBirmingham, ALUnited States; ^3^Kirchner GroupBirmingham, ALUnited States

**Keywords:** community health workers, mobile health, text messaging, cancer

## Abstract

**Background:**

Communication technologies can play a significant role in decreasing communication inequalities and cancer disparities by promoting cancer control and enhancing population and individual health. Studies have shown that technology, such as the mobile phone short message service (SMS) or text messaging, can be an effective health communication strategy that influences individuals’ health-related decisions, behaviors, and outcomes.

**Objective:**

The purpose of this study was to explore usage of communication technologies, assess the acceptability of mobile technology for delivery and access of health information, and identify cancer and health information needs among Deep South Network for Cancer Control trained Community Health Advisors as Research Partners (CHARPs).

**Methods:**

A mixed-method design was used, and a triangulation protocol was followed to combine quantitative and qualitative data. Focus groups (4 focus groups; n=37) and self-administered surveys (n=77) were conducted to determine CHARPs mobile phone and text message usage. The objective was to include identification of barriers and facilitators to a mobile phone intervention.

**Results:**

All participants were African American (37/37, 100%), 11/37 (89%) were women, and the mean age was 53.4 (SD 13.9; focus groups) and 59.9 (SD 8.7; survey). Nearly all (33/37, 89%) of focus group participants reported owning a mobile phone. Of those, 8/33 (24%) owned a smartphone, 22/33 (67%) had a text messaging plan, and 18/33 (55%) and 11/33 (33%) received and sent text messages several times a week or day, respectively. Similar responses were seen among the survey participants, with 75/77 (97%) reporting owning a mobile phone, and of those, 22/75 (30%) owned a smartphone, 39/75 (53%) had a text messaging plan, and 37/75 (50%) received and 27/75 (37%) sent text messages several times a week or day. The benefits of a text messaging system mentioned by focus group participants included alternative form of communication, quick method for disseminating information, and privacy of communication. The main barriers reported by both groups to using mobile technology to receive health information were cost and not knowing how to text message. Ways to overcome barriers were explored with focus group participants, and education was the most proposed solution. Majority of CHARPs were in favor of receiving a weekly text message that would provide cancer/health information.

**Conclusions:**

The findings from this study indicate that CHARPs are receptive to receiving text messages focusing on cancer/health information and would be likely to engage in mobile health research. These findings represent the first step in the development of an interactive mobile health program designed to provide cancer/health information and a support network for the Deep South Network Community Health Advisors as Research Partners (DSN CHARPs).

## Introduction

The American Cancer Society cites cancer as the second leading cause of death in the United States, with an estimated 1.7 million new cancer cases diagnosed and over half a million people expected to die of cancer in 2013 [1]. Studies have found that states in the southeastern United States exhibit much higher rates of new cancer diagnosis when compared with the national average. According to the most recent data, newly diagnosed cancer cases in the state of Alabama alone were expected to reach over 25,500, with lung, breast, prostate, and colorectal cancer being the four most common cancer types [2].

Cancer mortality has continued to decline in the United States in the past decade. However, certain racial and ethnic groups and people with low income and/or education continue to have the highest rates of both new cancers and cancer deaths [1]. African Americans have been shown to have the highest death rate and shortest survival of any racial and ethnic groups in the United States for most types of cancers [3]. In 2007, the death rate for all cancers combined continued to be 32% higher in African American men and 16% higher in African American women compared to Caucasian men and women, respectively [3]. Factors contributing to these disparities may include income, education, economic and social barriers to quality cancer prevention, and early detection and treatment services [3].

There are many diverse sources for seeking health information, with different populations and different ages using different strategies [4]. Studies have found people used personal sources, such as doctors, friends, and family members, to obtain most of their health information [5,6]. Gollop found that older African American women received health information from their physicians, the mass media, and members of their social networks [7]. James et al assessed preferences for seeking cancer information in the future among cancer patients, family/friends of cancer patients, and noncancer patients and found that all groups most frequently cited human sources (eg, health professionals and lay people) [8].

Communication technologies can play a significant role in decreasing communication inequalities and cancer disparities by promoting cancer control and enhancing population and individual health [9]. Mobile phones may be an appropriate means for addressing communication inequalities. Mobile phones are ubiquitous, and are becoming increasingly important in the delivery of health information. Mobile health (mHealth), as defined by the National Institutes of Health (NIH) Consensus group, is the use of mobile and wireless devices to improve health outcomes, health care services, and health research [10]. Furthermore, the short message service (SMS; also known as text messaging or texting) feature of the mobile phone is rapidly increasing in popularity. SMS allows for instantaneous delivery of messages to individuals at any time, place or setting, and are asynchronous, meaning messages can be accessed at any time by an individual [11]. The use of SMS permeates all age groups, cultures, and socioeconomic backgrounds [11]. According to a survey conducted in 2012 by the Pew Internet and American Life Project, 85% of US adults own a cell phone and 80% indicated they send and receive text messages [12]. People from racial/ethnic minorities were found to text message more than Caucasians, and African Americans were more likely than other cell phone owners to sign up for health text alerts and text with others in their neighborhood about community issues [12,13].

Recent studies have shown that text messaging can be an effective health communication strategy that influences individuals’ health-related decisions, behaviors, and outcomes [14-24]. There has been an increase in the development of health promotion programs that use the SMS feature of the mobile phone, either as an independent intervention, or in combination with other technologies or approaches. One approach used in health promotion programs is incorporating the assistance of community health advisors (CHAs). CHAs are lay community members who are seen as natural helpers and recognized by their friends, families, and neighbors as reliable sources of health information, help, and referrals [25]. Through formal training, CHAs help raise awareness, spread knowledge, and improve the health of their communities [25]. Studies have shown CHAs to be effective as they provide culturally appropriate, informal, and spontaneous assistance to community members [26]. Furthermore, they have been proven to be a useful method of overcoming barriers in the community because they are familiar with the local culture, local resources, and local health concerns [27].

The Deep South Network (DSN) for Cancer Control was created in early 2000 to establish a sustainable community infrastructure to promote cancer awareness among African Americans in the Alabama Black Belt and the Mississippi Delta [28]. To develop this infrastructure, “natural helpers,” who are defined as trusted and caring individuals who offer help to the community and/or its residents, were identified, recruited, and trained to become DSN Community Health Advisors as Research Partners (CHARP) [29]. The DSN program has trained and retained over 500 CHARPs in Alabama and Mississippi to educate and answer questions about breast, cervical, and colorectal cancer as well as address issues related to the health and health care access and resources in their community [30]. These CHARPs serve as a vital link between community members and community health agencies and resources. They bridge the gap between individuals and health care resources/cancer information by providing health education, explaining cancer screening tests, and enhancing community participation in clinical trials [29].

The development of an SMS-based program could enhance the CHARPs’ ability to reach a large number of people in the community and expand their outreach efforts with the convenience of using a mobile phone. The addition of an SMS system could support the delivery of accurate and up-to-date cancer health information through synchronous, real-time communication to community members. Furthermore, the SMS system could be used to overcome barriers in obtaining cancer health information across socioeconomic groups. In order to develop strategies to optimize the dissemination of cancer prevention and control information to CHARPs, formative research needs to be conducted to assess the feasibility and acceptability of a mobile phone-based program.

The purpose of this study was: (1) to explore the communication technologies used by the DSN CHARPs, (2) to assess the acceptability of an SMS-based mobile phone intervention, and (3) to identify cancer and health information needs.

## Methods

### Design

A two-part mixed methods study was conducted. First, the qualitative portion of the study involved focus groups conducted with DSN CHARPs in Alabama. Second, the quantitative, component involved collection of self-administered surveys completed by CHARPs in Alabama and Mississippi. The combination of qualitative and quantitative methodologies strengthened the interpretation of the data regarding feasibility and acceptability of using mobile phones for obtaining health information. By examining information collected by different methods and in different groups, the study findings can be corroborated across datasets, thereby reducing the impact of potential biases that can exist in a study [31].

### Study Population

Participants were recruited from DSN CHARPs residing in seven counties in Alabama and four counties in Mississippi. This study was approved by the Institutional Review Board at the University of Alabama at Birmingham.

### Data Collection

#### Focus Groups

DSN CHARPs from two Alabama counties were invited to participate in the focus groups through recruitment flyers, word-of-mouth, and the county coordinator. Four focus groups were conducted with a total of 37 participants, and the groups were divided by age (19-50 and 51 and older) based on evidence that different age groups may have different perspectives on technology [32]. At the start of each focus group, we obtained consent from the participants after study protocols and risks were explained. Following this, the participants completed a brief demographic and mobile phone usage questionnaire. The sessions lasted approximately 90 minutes, were digitally recorded, and were led by a trained moderator and co-moderator. Participants were paid $25 for their participation.

A semistructured interview protocol (see Multimedia Appendix 1) guided the discussion and ensured consistency between focus groups. The interview guide focused on current sources of cancer information, existing knowledge of cancer information and screening services, skills, and availability of resources relative to health information technology including mobile phones, feasibility and acceptability of using mobile phones to access health information, current use of mobile phones, knowledge and use of text messaging, and preference of information to be included in a text messaging database (eg, locations of cancer screenings based on zip code, free community cancer screenings). Participants were also asked what they perceive as barriers and facilitators to acceptability of a mobile phone intervention and what suggestions they may have for addressing the barriers.

#### County Survey

Based on a review of the literature and results from the focus groups, a 25-item questionnaire was developed by the study investigators with the purpose of assessing demographics, communication technologies usage, attitudes toward SMS usage, intention to use an SMS system, and social influences that predict SMS use. Questions included current cell phone ownership, type of cell phone (eg, iPhone, Blackberry, etc), text messaging plan (none, limited, unlimited), how often they send or receive text messages, purpose of text messages, and reasons for not using text messaging.

Survey packets were sent to the County Coordinators of nine DSN counties in Alabama (n=5) and Mississippi (n=4). The number of surveys sent was based on the number of active CHARPs within each county (n=119). A cover letter was attached to each survey. The cover letter included information about the research study, voluntary nature of participation, and a number to call if there were any questions about the study. By completing the survey, the individual agreed to participate in the study. The County Coordinators read aloud the cover letter and then distributed the surveys to the CHARPs during their monthly meeting. No identifiers were included on the survey other than the county.

### Analysis

The digitally recorded focus groups were transcribed to capture participant responses verbatim. The transcriptions were analyzed in two stages by qualitative content analysis [33]. In the first stage, two experienced coders independently read the original transcript and identified themes that were central to areas of discussion both within and across groups. Transcripts were coded using (1) codes and themes derived from the research questions and the moderator guide and (2) codes and themes that emerged from the data. Independent interpretations were discussed and the raters jointly decided upon a final coding scheme of relevant themes. The second stage of the analysis involved summarizing the data within and across groups and included a review of how the themes are interrelated. Themes that were considered relevant appeared within a topic of discussion by a minimum of three group members.

Descriptive analyses were used to report the demographic and mobile phone usage variables of the participants. Statistical analysis was carried out using SPSS version 20.

## Results

### Focus Groups

**Figure 1:**
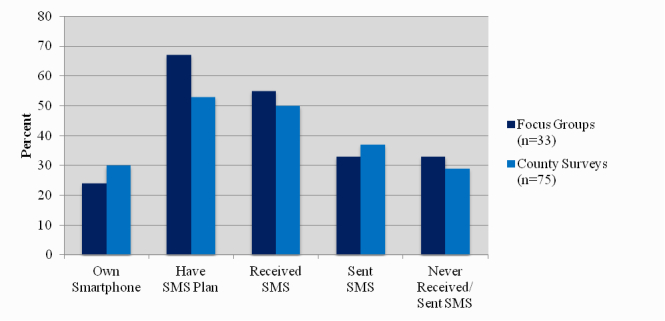
Smartphone ownership and SMS usage among participants owning a mobile phone.

Descriptive statistics were used to generate a profile of focus group participants based on demographic information (see Table 1). All participants were African American (37/37, 100%), 11/37 (89%) were women, mean age was 53.6 (SD 13.9), and 26/37 (70%) of participants had been a DSN CHARP for over 5 years. Nearly all participants reported owning a mobile phone (33/37, 89%). Of those, 8/33 (24%) indicated they had a smartphone (eg, Blackberry, iPhone) and 22/33 (67%) had a texting plan (limited/unlimited). A total of 18/33 (55%) received text messages either several times a week (7/33, 21%) or day (11/33, 33%), and 11/33 (33%) sent text messages either several times a week (2/33, 6%) or day (9/33, 27%) (see Figure 1).

### County Survey

A total of 77/119 surveys were completed for a response rate of 65%. All participants were African American (77/77, 100%), 69/77 (90%) were women, the mean age was 59.9 (SD 8.7), and 62/77 (81%) of participants had been a DSN CHARP for over 5 years (see Table 1). Similar to the focus groups, nearly all participants reported owning a cell phone (75/77, 97%), and of those 22/75 (30%) indicated they had a smartphone (eg, Blackberry, iPhone) and 39/75 (53%) had a texting plan (limited/unlimited). A total of 37/75 (50%) received text messages several times a week (20/75, 27%) or day (17/75, 23%), and 27/75 (37%) sent text messages several times a week (14/75, 19%) or day (13/75, 18%), and 22/75 (29%) never sent or received a text message (see Figure 1).

### Qualitative and Quantitative Results

#### General

The major themes that were identified from the focus group discussions are summarized in Table 2. The telephone was reported by focus group participants as the most common method community members used to contact CHARPs for health information. In-person and telephone were the most common methods community members used to contact CHARPs for health information as reported by survey participants.

**Table 1:** Focus group and county survey participants’ demographic information.

Variable		Focus group^a^	County survey^b^
		n=37	n=77
**Gender, n (%)**			
	Female	33 (89)	69 (90)
	Male	4 (11)	8 (10)
**Age**			
	Mean (SD)	53.4 (13.9)	59.9 (8.7)
	Range	25-79	36-84
Race, African American, n (%)		37 (100)	77 (100)
**Marital status, n (%)**			
	Married	17 (46)	28 (36)
	Widowed/Divorced/Separated	12 (32)	37 (48)
	Never Married	8 (22)	11( 14)
**Educational level, n (%)**			
	Less than high school	7 (19)	5 (6)
	High school or GED	14 (38)	12 (16)
	Some college	8 (22)	26 (34)
	Bachelor’s degree or higher	8 (22)	34 (44)
**Employment, n (%)**			
	Employed	19 (52)	39 (50)
	Retired	12 (32)	23 (30)
	Other^c^	5 (13)	15 (20)
Has children < 18 years old, n (%)		15 (41)	19 (25)
CHARPs with DSN > 5 years, n (%)		26 (70)	62 (81)

^a^One missing value for employment.

^b^One missing value for marital status.

^c^Other = homemaker, unable to work, out of work.

**Table 2:** Focus group themes.

Theme			Number of comments (%)
**Request information (n=20)**			
	Cancer information		6 (30)
	Low-cost/free health care services		5 (25)
	Transportation services		2 (10)
	Miscellaneous		7 (35)
**Text messaging**			
	**Facilitators for text messaging (n=18)**		
		Alternate form of communication	7 (39)
		Quickness	5 (28)
		Personal	3 (17)
		Privacy	3 (17)
	**Facilitators for text messaging to receive health information (n=16)**		
		Dissemination of information	6 (38)
		Awareness	4 (25)
		Other^a^	6 (38)
	**Barriers for text messaging (n=21)**		
		Not knowing how to text	6 (29)
		Cost	5 (24)
		Confidentiality	4 (19)
		Other^b^	6 (29)

^a^Other = prevention, communication, quickness, popular.

^b^Other = age, no reason to text, spam text messages, multiple messages.

#### Requested Information

Focus group participants mentioned low-cost or free health care services, cancer information (eg, screening, general information, survivorship), and transportation services as the most commonly requested information. For example:

I get calls from family members, like questions about different health problems especially about high blood pressure, diabetes, diabetic.

I have friends calling asking me for information about the free services, if they don’t have the money to have a mammogram.

Anywhere they can go and you know have screening done for free or do you know of anybody that could give a discount or something, transportation.

For me they want to know how did I felt when I first found out, you know, I had cancer and how did I handle it, you know, during that period and until after, you know, my breast was removed.

I have friends call and ask me about colonoscopy, a lot of people afraid to take it and when they ask me I tell them there’s nothing to it, there’s not.

Survey participants’ most commonly requested information included breast cancer, nutrition, and physical activity.

#### Text Messaging

##### Facilitators for Text Messaging

Focus group participants identified alternate form of communication (7/18, 39%), quickness (5/18, 28%), personal (3/18, 17%), and privacy (3/18, 17%) as the main reasons for text messaging.

It’s a quick response to a question, to get information to a person.

You don’t have to worry about if you’re in a crowded place and somebody else hears your conversation.

This is another way of having close contact with a person, you know. They have unlimited calling, you know, but a lot of times now you can save the amount of time on the phone by just using your text.

Sometimes you’re in a place where you can’t talk to somebody or you need to send a message to somebody and you don’t. You need to be quiet and you need to get a message out.

Has a certain degree of privacy.

Survey participants reported personal (46/75, 62%) and work-related (20/75, 26%) communications were the main reasons for sending/receiving text messages.

##### Facilitators for Text Messaging to Receive Health Information

Focus group participants also mentioned the ability to disseminate information (6/16, 38%) and awareness (4/16, 25%) as key reasons for using text messaging to receive health information.

Then you can immediately get them information of where they need to go I also think that text messaging, like you said, it’s to help each other. If you have some friends out there in the community that you know the phone number, you could now get the information out in different homes, you know, if they have cell phones. Even though you might have people’s numbers and just spread the news about cancer, you know, in the community if you know the people’s phone numbers.

Maybe if your family needs to go, like if say for instance you text me something, and I can text them to everybody in my phone. I can just forward it to everyone, and so you forward it to me and I could forward it to ten more. They could forward it to ten more, and so there are 20 people that have been told, you know?

##### Barriers for Text Messaging

Cost, not knowing how to text, and confidentiality were the most frequently mentioned barriers to texting. For example:

I don’t know the first thing about it.

I don’t know how to and I’ve never taken the time to learn.

Of course one for most people would be not having a plan because if you don’t have a plan it could get costly.

I would think not having the texting knowledge or know-how.

Maybe someone else might see the information on the phone, and they really didn’t want anybody else to see it. No cell phone.

Survey participants’ most frequently mentioned barriers to texting included: cost (23/77, 30%), not knowing how to text (11/77, 15%), and not wanting to receive too many messages (21/77, 27%).

Almost half of the focus group participants (9/20, 45%) viewed education as the best solution to overcome barriers. The majority of the focus group participants and nearly half of the survey respondents were in favor of receiving a weekly text message that provided cancer/health information.

## Discussion

### Principal Findings

Our findings suggest that DSN CHARPs are receptive to the use of mobile technology for access and delivery of information, such as health issues (eg, cancer, nutrition, and physical activity), location of health care services, and availability of local transportation services. Furthermore, they are likely to engage in mHealth research because texting provides an alternative form of communication and dissemination as well as social support. To date, there is only one study that has identified projects utilizing mobile technologies for community health workers [34]. Kallander et al [34] conducted a thematic review with the focus on low- and middle-income countries. They found only a limited number of mHealth projects and very few formal outcome evaluations.

The combination of qualitative and quantitative data collection and analysis methods allowed for a rich understanding of the role of DSN CHARPs in the community and their perceived barriers and facilitators to mobile technology. Although the cost of texting and not knowing how to text were the most frequently cited barriers to an SMS-based program, over half of the CHARPs had a text messaging plan (limited or unlimited). In order to overcome barriers, participants indicated they can be taught how to access and read the texts, which is similar to a study conducted by Pfaeffli et al [32] that found participants in their senior years were interested in taking part in a mobile phone-based exercise intervention, but said they would need assistance. Therefore, participants were taught how to open texts, view messages, and assistance was provided to those who encountered difficulties. These results show that barriers to an SMS-based program can be overcome by providing education to those initially reluctant to texting.

Majority of CHARPs have been volunteers for the DSN Program for over five years, and have been exposed to and have adopted different methods to deliver health information, such as local media messages, town hall meetings, cancer awareness walks, and one-on-one meetings [30]. The addition of mobile technology as a tool has potential to support the performance of CHARPs by disseminating information and prevention messages and by directing individuals to local health care services. It can also be used to keep CHARPs informed of upcoming community events and of recent developments in health or cancer research. Furthermore, CHARPs can communicate directly with one another and provide social support. Our findings demonstrate an important role in developing an SMS-based program as a tool to support community health workers. The next steps are to collaborate with the CHARPs to develop and evaluate a culturally relevant SMS-based project.

### Limitations

This study has some limitations. First, the view of African Americans in the rural Deep South may not be generalizable to those in urban areas. However, research has shown that African Americans are more likely to sign up for health text alerts as well as text with others in their neighborhood about community issues [12,13]. Second, the focus group discussion and the county survey may have elicited different responses. Although the questions for both the focus group and county survey were written in the second person, focus group participants answered questions in reference to both themselves and others they knew, whereas the county survey participants most likely answered questions in reference only to themselves. Also, the focus of this study was on SMS use, so there may be opportunities to explore other information and communication technologies, such as social media, as this is constantly growing and ever-changing field [35].

### Conclusions

The findings from this study represent the first step in the development of an interactive mobile health program designed to provide cancer/health information and a support network for the DSN CHARPs. This program could be used to increase the accessibility of cancer/health information, thereby increasing the effectiveness of the DSN CHARPs. Health disparities exist not only in health care but also in the ability of different population groups to access and use health information. In order to reach population subgroups with important health information, it is necessary to use the channels through which they seek such information. Our findings suggest the combination of CHARPs and mobile technology may improve access and delivery of information and services, particularly for rural and underserved populations.

Future studies are needed to determine the acceptance of receiving and accessing cancer health information and participating in studies related to mobile research with community health workers beyond low- and middle-income countries.

## Multimedia Appendix 1

Focus group topic guide.

## Abbreviations

CHAs: community health advisors
CHARPs: community health advisors as research partners
DSN: deep south network
mHealth: mobile health
NIH: national institutes of health
SMS: short message service

## References

[1] American Cancer Society (2013). Cancer Facts & Figures 2013.

[2] Alabama Statewide Cancer Registry (2012). Alabama Cancer Facts & Figures 2011.

[3] American Cancer Society (2011). Cancer Facts & Figures for African Americans 2011-2012.

[4] Muha C, Smith KS, Baum S, Ter Maat J, Ward JA (1998). The use and selection of sources in information seeking: the Cancer Information Service experience. Part 8. J Health Commun.

[5] Buller DB, Callister MA, Reichert T (1995). Skin cancer prevention by parents of young children: health information sources, skin cancer knowledge, and sun-protection practices. Oncol Nurs Forum.

[6] Cangelosi JD, Markham FS (1994). A descriptive study of personal, institutional, and media sources of preventive health care information. Health Mark Q.

[7] Gollop CJ (1997). Health information-seeking behavior and older African American women. Bull Med Libr Assoc.

[8] James C, James N, Davies D, Harvey P, Tweddle S (1999). Preferences for different sources of information about cancer. Patient Educ Couns.

[9] Viswanath K, Nagler RH, Bigman-Galimore CA, McCauley MP, Jung M, Ramanadhan S (2012). The communications revolution and health inequalities in the 21st century: implications for cancer control. Cancer Epidemiol Biomarkers Prev.

[10] National Institutes of Health Consensus Group mHealth.

[11] Fjeldsoe BS, Marshall AL, Miller YD (2009). Behavior change interventions delivered by mobile telephone short-message service. Am J Prev Med.

[12] Fox S, Duggan M (2012). Mobile Health 2012.

[13] Smith A (2010). Neighbors Online.

[14] Hurling R, Catt M, Boni MD, Fairley BW, Hurst T, Murray P, Richardson A, Sodhi JS (2007). Using internet and mobile phone technology to deliver an automated physical activity program: randomized controlled trial. J Med Internet Res.

[15] Rutten LJ, Squiers L, Hesse B (2006). Cancer-related information seeking: hints from the 2003 Health Information National Trends Survey (HINTS). J Health Commun.

[16] Franklin V, Waller A, Pagliari C, Greene S (2003). "Sweet Talk": text messaging support for intensive insulin therapy for young people with diabetes. Diabetes Technol Ther.

[17] Franklin VL, Greene A, Waller A, Greene SA, Pagliari C (2008). Patients' engagement with "Sweet Talk" - a text messaging support system for young people with diabetes. J Med Internet Res.

[18] Franklin VL, Waller A, Pagliari C, Greene SA (2006). A randomized controlled trial of Sweet Talk, a text-messaging system to support young people with diabetes. Diabet Med.

[19] Joo NS, Kim BT (2007). Mobile phone short message service messaging for behaviour modification in a community-based weight control programme in Korea. J Telemed Telecare.

[20] Kwon HS, Cho JH, Kim HS, Lee JH, Song BR, Oh JA, Han JH, Kim HS, Cha BY, Lee KW, Son HY, Kang SK, Lee WC, Yoon KH (2004). Development of web-based diabetic patient management system using short message service (SMS). Diabetes Res Clin Pract.

[21] Kollmann A, Riedl M, Kastner P, Schreier G, Ludvik B (2007). Feasibility of a mobile phone-based data service for functional insulin treatment of type 1 diabetes mellitus patients. J Med Internet Res.

[22] Kim HS (2007). A randomized controlled trial of a nurse short-message service by cellular phone for people with diabetes. Int J Nurs Stud.

[23] Cole-Lewis H, Kershaw T (2010). Text messaging as a tool for behavior change in disease prevention and management. Epidemiol Rev.

[24] Rodgers A, Corbett T, Bramley D, Riddell T, Wills M, Lin RB, Jones M (2005). Do u smoke after txt? Results of a randomised trial of smoking cessation using mobile phone text messaging. Tob Control.

[25] Johnson RE, Green BL, Anderson-Lewis C, Wynn TA (2005). Community health advisors as research partners: an evaluation of the training and activities. Fam Community Health.

[26] Martin MY (2005). Community health advisors effectively promote cancer screening. Ethn Dis.

[27] Martin MY, Kim YI, Kratt P, Litaker MS, Kohler CL, Schoenberger YM, Clarke SJ, Prayor-Patterson H, Tseng TS, Pisu M, Williams OD (2011). Medication adherence among rural, low-income hypertensive adults: a randomized trial of a multimedia community-based intervention. Am J Health Promot.

[28] Partridge EE, Fouad MN, Hinton AW, Hardy CM, Liscovicz N, White-Johnson F, Higginbotham JC (2005). The deep South network for cancer control: eliminating cancer disparities through community-academic collaboration. Fam Community Health.

[29] Hardy CM, Wynn TA, Huckaby F, Lisovicz N, White-Johnson F (2005). African American community health advisors trained as research partners: recruitment and training. Fam Community Health.

[30] Lisovicz N, Johnson RE, Higginbotham J, Downey JA, Hardy CM, Fouad MN, Hinton AW, Partridge EE (2006). The Deep South Network for cancer control. Building a community infrastructure to reduce cancer health disparities. Cancer.

[31] Jick TD (1979). Mixing qualitative and quantitative methods: triangulation in action. Administrative Science Quarterly.

[32] Pfaeffli L, Maddison R, Whittaker R, Stewart R, Kerr A, Jiang Y, Kira G, Carter K, Dalleck L (2012). A mHealth cardiac rehabilitation exercise intervention: findings from content development studies. BMC Cardiovasc Disord.

[33] Patton MQ (2002). Qualitative research and evaluation methods.

[34] Källander K, Tibenderana JK, Akpogheneta OJ, Strachan DL, Hill Z, ten Asbroek AH, Conteh L, Kirkwood BR, Meek SR (2013). Mobile health (mHealth) approaches and lessons for increased performance and retention of community health workers in low- and middle-income countries: a review. J Med Internet Res.

[35] Abroms LC, Ahuja M, Kodl Y, Thaweethai L, Sims J, Winickoff JP, Windsor RA (2012). Text2Quit: results from a pilot test of a personalized, interactive mobile health smoking cessation program. J Health Commun.

