# Supplementary material for: Acceptability of Delivering and Accessing Health Information Through Text Messaging Among Community Health Advisors
Source: JMIR Mhealth Uhealth. 2013 Sep 9;1(2):e22. doi: 10.2196/mhealth.2641 (PMC4114512; doi:10.2196/mhealth.2641)
Supplement: Supplementary file 1 [file mhealth_v1i2e22_app1.pdf]

Multimedia Appendix 1

Focus Group Topic Guide

INTRODUCTION

- Explain Process: *Note takers will manually record your responses. No identifying information will be linked to your responses.*
- Explain Ground Rules: *There are no right or wrong answers, only your honest opinions are requested. Please respect each other's opinions; all opinions are important. Although you may be the only person in the group holding a particular opinion, you may represent a significant point of view in the community. Finally, please speak up and speak one-at-a-time. We look forward to everyone's participation.*

- *I'd like to begin today by having each of you tell me a little about yourself.*

a. First name and anything you would like to share about yourself
b. Tell me about the type of cell phone you have (regular, Blackberry, Windows-based phone (PDA), iPhone)?

QUESTIONS

1. As a CHARP, how often do community members ask you for cancer information?
    a. What are the most common methods people contact you for cancer information (phone, in-person, email)?
    b. How you do prefer to be contacted?
    c. What are the most common questions asked?

2. How often have you been asked a question about a cancer topic you do not know the answer to?
    a. Where do you go first to find the information?
    b. What type of information were you looking for (specific cancer screening tests, symptoms of cancer, where to get a low cost/free screening)?
    c. What difficulties did you have in finding the information you needed?
    d. What facilitated getting the information you needed?
    e. How satisfied were you with the quality of the information?

3. Thinking about how you get cancer information and communicate with others…What types of technology do you think would be helpful in getting cancer information when you need it? (internet – email, website; phone – interactive voice response system, cell phone – video)
    a. How about receiving cancer information? (Information provided when you are not looking for it)

4. Other than using technology, what other ways do you think would be helpful in getting cancer information when you need it?
    a. How about receiving cancer information? (Information provided when you are not looking for it)

5. A method for obtaining cancer information we would like to explore is text messaging. Text messaging is a way to communicate on your cell phone by sending a written message from one cell phone to another using your phone's keypad.
    a. Have you ever used text messaging to communicate with someone?
    PROBE: If <u>YES</u>, How often? What are the reasons you use text messaging? What do you use it for?
    If <u>NO</u>, what are the reasons?

6. Now, I would like to hear your thoughts on whether this would be a way to get cancer information when you need it (information needs to be looked up).
    a. How do you think this could be useful?
    b. What would be the drawbacks?
    c. What do you think you would use it for?
    d. What would be the reasons for not using text messaging for getting cancer information?
    e. What would be problems for using text messaging to get cancer information?
    f. What can be done to overcome barriers?

7. What do you think about receiving a weekly text message which provides cancer information such as dates and locations of free cancer screenings or simple cancer tips?

Focus Group Topic Guide

8. Is there anything else that you would like to share that would be helpful?
